# Screening for Early Endometriosis Detection (SEED): Co-development of a Symptom-Based Screening Measure for Canadian Young Adults in Primary Care

**DOI:** 10.1080/24740527.2026.2729539

**Published:** 2026-10-15

**Authors:** Rachael Bosma, Brittany N. Rosenbloom, Adriano Nella, Lindsay Wolfson, Mathew Leonardi, Magali Robert, M. Jonathon Solnik, Arun Radhakrishnan, Katelynn E. Boerner, Philippa Bridge-Cook, Katelyn Luciani, Tania Di Renna

**Affiliations:** a Toronto Academic Pain Medicine Institute (TAPMI), Women’s College Hospital, Toronto, Ontario, Canada; b Department of Anesthesiology and Pain Medicine, University of Toronto, Toronto, Ontario, Canada; c Centre of Excellence for Women’s Health, Vancouver, British Columbia, Canada; d Department of Obstetrics and Gynecology, McMaster University, Hamilton, Ontario, Canada; e Department of Obstetrics and Gynecology, Department of Anesthesiology, Perioperative and Pain Medicine, Cumming School of Medicine, Calgary, Alberta, Canada; f Department of Obstetrics and Gynecology, University of Toronto, Toronto, Ontario, Canada; g Department of Obstetrics and Gynecology, Sinai Health System, Toronto, Ontario, Canada; h Department of Family Medicine, University of Ottawa, Ottawa, Ontario, Canada; i Bruyere Health Research Institute, Ottawa, Ontario, Canada; j Department of Pediatrics, BC Children’s Hospital Research Institute, University of British Columbia, Vancouver, British Columbia, Canada; k The Endometriosis Network Canada, Toronto, Ontario, Canada

**Keywords:** Endometriosis, screening tool, screening measure, Delphi study, gynecologyF, primary care, chronic pelvic pain

## Abstract

**Background:**

Many Canadians experience prolonged pain, uncertainty, and stigma before receiving an endometriosis diagnosis, with delays averaging 11 years from symptom onset. Endometriosis, defined by the presence of endometrial-like tissue outside the uterine cavity, impacts 10% of Canadian women and approximately 9% of transgender men. Existing screening measures are limited because they often focus on advanced disease and were developed outside Canada.

**Aims:**

The aim of this study is to revise the ENDOPAIN-4D screening measure to match a Canadian young adult (age 18–25) population.

**Methods:**

Using a participatory approach, the study involved four phases: an initial Delphi consensus survey with Canadian endometriosis experts; a virtual working meeting with a purposively selected subgroup; a second Delphi survey with working group members to refine proposed changes and reach consensus; and a third Delphi to collect final feedback. Participants included people with lived and living experience of endometriosis, gynecologists, pain medicine specialists focused on chronic pelvic pain, primary care providers, and other healthcare professionals involved in endometriosis care.

**Results:**

Sixty-five experts participated in the first Delphi round. Five major themes were identified: conceptual appropriateness of domains, question clarity, response option clarity, item redundancy, and simplification for a Canadian young adult audience. Fifteen experts participated in the working meeting and second Delphi round, where they refined item wording, clarified core concepts, and finalized items for the revised screening measure.

**Conclusions:**

This study adapted the ENDOPAIN-4D for Canadian young adults, resulting in a 22-item, five-domain screening measure called Screening for Early Endometriosis Detection (SEED).

## RÉSUMÉ

**Contexte :** Au Canada, de nombreuses personnes vivent avec des douleurs persistantes, de l’incertitude et de la stigmatisation avant de recevoir un diagnostic d’endométriose, le délai moyen entre l’apparition des symptômes et le diagnostic étant de onze ans. L’endométriose, définie par la présence de tissu semblable à l’endomètre en dehors de la cavité utérine, touche 10 % des femmes canadiennes et environ 9 % des hommes transgenres. Les outils de dépistage existants présentent des limites, car ils ciblent souvent les formes avancées de la maladie et ont été élaborés à l’extérieur du Canada.

**Objectifs :** L’objectif de cette étude est d’adapter l’outil de dépistage ENDOPAIN-4D à une population canadienne de jeunes adultes âgés de 18 à 25 ans.

**Méthodes :** L’étude, menée selon une approche participative, comportait quatre phases : une première enquête Delphi auprès d’experts canadiens de l’endométriose visant à établir un consensus; une réunion de travail virtuelle avec un sous-groupe sélectionné de manière raisonnée; une deuxième enquête Delphi auprès des membres du groupe de travail pour affiner les modifications proposées et parvenir à un consensus ; et une troisième enquête Delphi pour recueillir les derniers commentaires. Les participants comprenaient des personnes ayant une expérience passée ou actuelle de l’endométriose, des gynécologues, des spécialistes de la médecine de la douleur se consacrant aux douleurs pelviennes chroniques, des prestataires de soins de première ligne et d’autres professionnels de la santé participant à la prise en charge de l’endométriose.

**Résultats :** Soixante-cinq experts ont participé au premier tour Delphi. Cinq thèmes principaux ont été dégagés : la pertinence conceptuelle des domaines, la clarté des questions, la clarté des options de réponse, la redondance des énoncés et la simplification pour un public canadien de jeunes adultes. Quinze experts ont participé à la réunion de travail et au deuxième tour Delphi, au cours desquels ils ont affiné la formulation des énoncés, clarifié les concepts fondamentaux et finalisé les énoncés de la version révisée de l’outil de dépistage.

**Conclusions :** Cette étude a adapté l’ENDOPAIN-4D aux jeunes adultes canadiens, aboutissant à un outil de dépistage comportant 22 énoncés répartis en cinq domaines, appelé *Screening for Early Endometriosis Detection* (SEED).

## Introduction

Despite decades of research aimed at improving the diagnosis of endometriosis, timely identification in routine clinical care remains uncommon.^1–3^ Many Canadians face 11 years of debilitating pain, uncertainty, anxiety, depression, and stigma on their journey from symptom onset to a clinical diagnosis of endometriosis.^4^ Endometriosis is a systemic inflammatory condition characterized by the presence of endometrial-like tissue outside the uterine cavity and impacts roughly 10% of Canadian women and approximately 8.9% of transgender men.^5–7^ Endometriosis has substantial impacts on quality of life, physical functioning, and mental health.^8,9^

This prolonged diagnostic delay reflects a persistent gap between what is known about endometriosis symptoms and how this knowledge is recognized and acted upon in everyday clinical practice. Most women report seeing a doctor a minimum of five times before receiving a diagnosis or being referred to a gynecologist for further diagnostic evaluation,^10^ despite nearly two thirds reporting symptoms for several years, often beginning in adolescence.^11^ The incidence of this diagnostic delay is multifactorial, with the societal normalization of menstrual pain, nonspecific or heterogeneous symptoms, limited disease awareness from healthcare providers and patients, and a historical reliance on invasive measures (e.g., surgery) for disease confirmation contributing heavily to the disease burden.^6,12–15^ As a result, an estimated 60% of endometriosis cases remain undiagnosed, underscoring the need for strategies that support earlier recognition and clinical action on symptom presentation in frontline care settings.^16^

Symptom-based screening measures represent a key mechanism for translating evidence into clinical decision-making. Recent guidelines recommend the use of noninvasive, symptom-focused diagnosis^17,18^ commonly including specialized ultrasound and magnetic resonance imaging.^19,20^ Regardless, these methods are not available in primary care settings resulting in a lengthy and traumatizing loop of appointments and referrals to multiple specialists before achieving diagnosis. The appropriate screening of people reporting symptoms related to endometriosis and subsequent referral to a gynecologist to confirm diagnosis would streamline this process, support primary care providers, and enhance access to timely care for patients. People living with endometriosis and advocacy groups have identified developing a noninvasive screening measure to aid in early detection of endometriosis as one of their top priorities.^21^

A recent scoping review identified the ENDOPAIN-4D as the strongest available endometriosis screening measure, demonstrating high reliability, validity, and sensitivity for early detection.^22^ However, most screening measures, including the ENDOPAIN-4D, have been developed and validated in adult populations with advanced disease. The ENDOPAIN-4D was validated in a sample with a mean age of 38.8 years and outside the Canadian primary care context, raising concerns about its relevance and utility for younger populations and earlier stages of symptoms. Endometriosis may present differently across the lifespan, highlighting the need for developmentally appropriate screening measures for young adults, with items that reflect age-relevant priorities.^23–25^ In addition, screening measures must be cross-culturally validated to ensure applicability in the Canadian context and evaluated for gender sensitivity, using trauma-informed design, and non-stigmatizing language.

The aim of this study is to co-create with gynecologists, primary care providers (PCPs), people with lived/living experience (PWLE), and pain specialists, an English version of a valid, evidence-informed, and gender-inclusive symptom-based screening measure to support the early identification of endometriosis in young adults presenting to primary care in Canada. Guided by the Knowledge-to-Action framework,^26^ we assessed and refined the content validity (i.e. the degree to which the measure accurately reflects the target construct,^27^ which in this study was the presence of an endometriosis diagnosis) of an existing symptom-based screener and, through an iterative, expert-informed process, adapted and co-developed a Canadian-contextualized measure for use in frontline clinical settings, where diagnostic uncertainty is common and access to specialized testing is limited. Input from gynecologists, pain medicine specialists, primary care clinicians, researchers, sex and gender experts, and individuals with lived and living experience of endometriosis was integrated to ensure the screener reflects clinically meaningful symptom patterns, patient priorities, and the practical constraints of primary care.

## Materials and methods

This study was informed by a participatory research approach. Three individuals with lived experience of endometriosis and chronic pain were members of the study team. They contributed to the conceptualization of the study, refinement of research questions, interpretation of Delphi findings, and development of revisions to the screening measure. Their involvement helped ensure that the measure reflects patient priorities, lived symptom experiences, and considerations related to stigma, accessibility, and real-world usability in primary care settings.

Guided by this participatory approach, we conducted a modified Delphi process with a working meeting to revise an endometriosis screening measure targeting young adults aged 18–25 years in Canada (see Figure 1). Delphi methods often involve multiple rounds of expert feedback to reach consensus and have been widely applied in clinical research to develop and refine questionnaires and measures.^28–30^ This study comprised four phases: (1) an initial Delphi consensus survey distributed to a broad group of Canadians with expertise in endometriosis (clinicians, researchers, people with lived/living experience), (2) a virtual working meeting with a purposively selected subgroup of relevant experts, (3) a subsequent Delphi consensus survey conducted with members of the working group to refine modifications and achieve consensus, and (4) a third Delphi round to collect agreement on the final screening measure. The study was approved by the Women’s College Hospital ethics committee (REB # 2024–0065-E).

**Figure 1.**
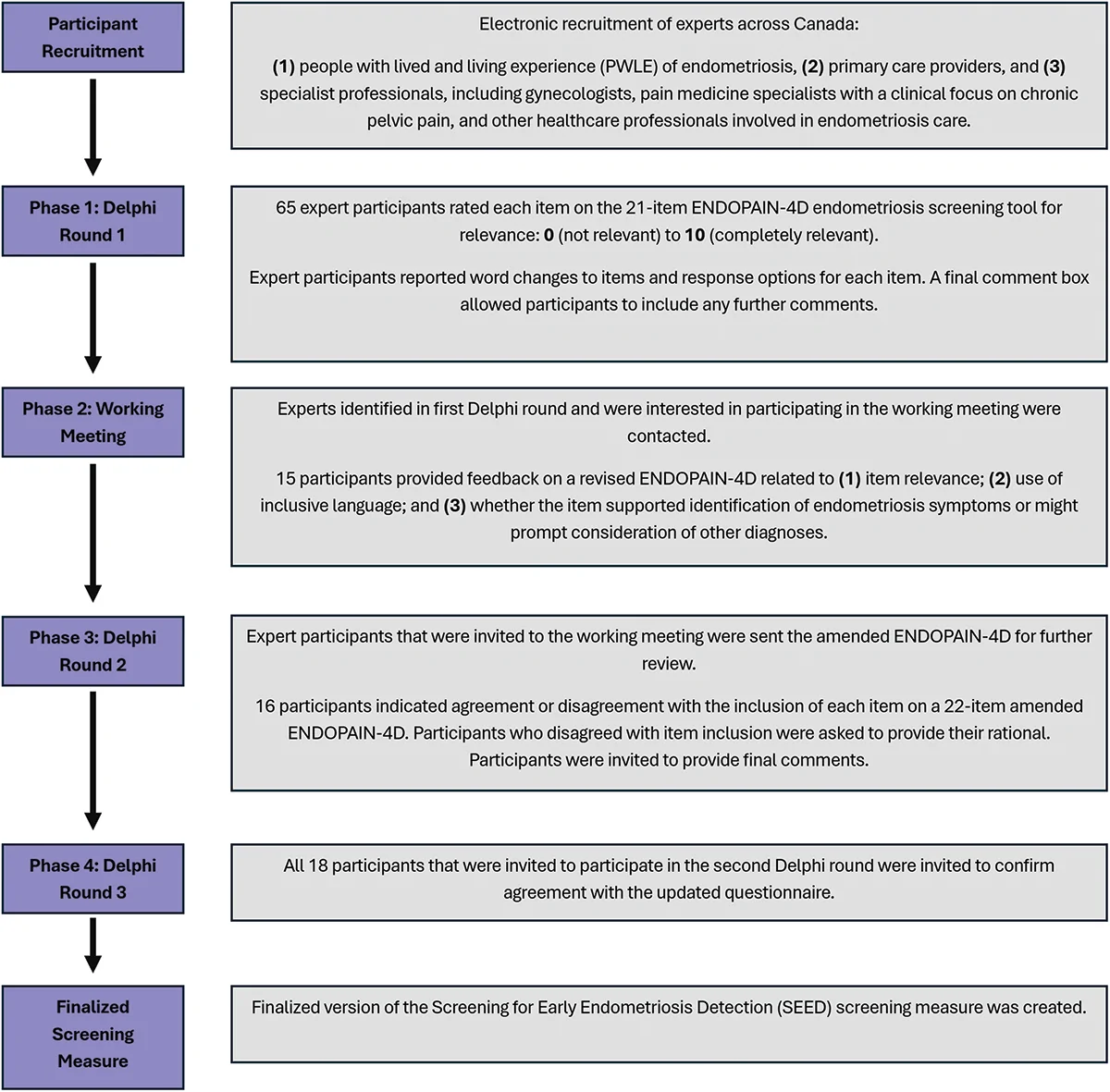
Delphi flow diagram.

### Recruitment

The Delphi panel comprised three intended interest-holder groups: (1) people with lived and living experience (PWLE) of endometriosis, (2) primary care providers, and (3) specialist professionals, including gynecologists, pain medicine specialists with a clinical focus on chronic pelvic pain, and other healthcare professionals involved in endometriosis care.

For the PWLE group, we sought perspectives from individuals who were currently aged 18 to 25 years, as well as individuals who were older at the time of participation and who had experienced endometriosis symptoms, diagnostic assessment, or diagnosis between the ages of 18 and 25. The latter group was included because their retrospective accounts could provide relevant insight into symptom experiences and barriers to recognition and diagnosis during the developmental period for which the screening measure was intended. While there is no universally accepted standard size for a Delphi panel, we aimed to ensure diverse representation across geographic regions and varying levels of experience in endometriosis care and research. We, therefore, targeted approximately 60 participants (20 from each expert group; specialists, PCP, PWLE), a sample size that is generally considered optimal for a relatively homogenous Delphi panel, balancing diversity of expertise with feasibility and meaningful consensus generation.^31^ Participants were identified through professional and community networks, including patient advocacy and education organizations (e.g., The Endometriosis Network Canada), as well as academic registries and personal professional connections. Potential participants were contacted via e-mail and invited to participate in the study.

At the conclusion of the first Delphi survey round, participants were asked to indicate their willingness to participate in a subsequent expert working group to further adapt the screening measure based on survey findings. From those who expressed interest, we used purposive sampling to assemble an expert working group with representation across the three interest-holder groups: people with lived and living experience of endometriosis, primary care providers, and specialist professionals. Within and across these groups, we also sought diversity in clinical discipline and expertise, including gynecology, chronic pain medicine/psychology, neurodiversity, and sex and gender research. This ensured a range of perspectives to support in-depth discussion and refinement of the questionnaire. Because feedback from Delphi Round 1 resulted in substantial changes that extended beyond minor adaptation of the original measure, subsequent phases focused on detailed co-development and refinement with this purposively selected working group. The same group attended the virtual working meeting and participated in Delphi Rounds 2 and 3.

### Phase 1: Delphi Round 1

The first Delphi round was conducted electronically via e-mail, during which participants reviewed the items from the ENDOPAIN-4D for relevance, comprehensiveness, and comprehensibility.^27,32^ Item review occurred in two stages. First, clinical relevance was assessed quantitatively. Participants rated the relevance of each item for screening for endometriosis among Canadian young adults on a scale from 0 (not relevant) to 10 (completely relevant). For each item, the mean relevance score across all participants was calculated and converted to a percentage of the maximum possible score by dividing the mean score by 10 and multiplying by 100. A priori, items were required to achieve a percentage relevance score greater than 75% for inclusion in the revised screening measure.^33^ Items not meeting this threshold with a percentage relevance score of 75% or lower were considered for removal. This threshold was applied across the full participant panel and was not assessed separately within the three interest-holder groups. The mean-based threshold was retained because it was the prespecified decision rule used during the Delphi process. As the distributions of the individual relevance ratings were skewed toward the upper end of the scale, item ratings are descriptively reported using both the mean and standard deviation as well as the median and interquartile range. Respondents were further asked to comment on clarity and wording (comprehensiveness and comprehensibility) by providing wording changes to items and response options for each item of the ENDOPAIN-4D. A final open comment box allowed participants to propose additional amendments.

Second, items meeting the relevance threshold underwent qualitative review by the same experts to assess content validity with a focus on face validity (i.e. the degree that the items on the measure look to be an adequate reflection of the construct being measured^27^) within the Canadian context. This stage examined responses indicating “yes, with modification” for both item wording and response options. No predefined numerical threshold was applied for modification. This approach was intentional, as the objective was not only to retain clinically relevant constructs but also to ensure that language was inclusive, non-stigmatizing, developmentally appropriate for young adults, and feasible for primary care use.

Qualitative data consisted of open-text comments and suggested revisions submitted by participants. An inductive content analysis approach was used to group comments into content categories related to item relevance, comprehensiveness, and comprehensibility.^34^ Three members of the research team (AN, RB, BR) independently reviewed and coded qualitative responses, with discrepancies resolved through discussion to achieve consensus. Particular attention was paid to feedback concerning feasibility and usability in primary care settings.

All items for which modifications were recommended were subsequently reviewed in detail during the expert working meeting. Findings from the qualitative analysis directly informed revisions to item wording and response options in subsequent Delphi rounds. This iterative process ensured that retained items met predefined relevance criteria while also achieving contextual appropriateness for Canadian primary care settings.

### Phase 2: Working meeting

During the working meeting with experts identified from Phase 1, each item was reviewed systematically. For every item, participants were presented with (1) the original ENDOPAIN-4D item, (2) a summary of quantitative results and qualitative feedback from Delphi Round 1, and (3) a proposed revised version developed by the research team based on the initial analysis. This structured approach facilitated focused discussion and transparent decision-making.

Participants were asked to provide feedback across three domains: (1) item relevance for the Canadian context; (2) the appropriateness, accessibility, and non-stigmatizing nature of the language used; and (3) whether the item supported identification of symptom patterns consistent with and specific to endometriosis or alternatively might prompt consideration of other diagnoses. Inclusive language use considered gender, neurodiversity, fertility wishes, cultural references/examples, sexual orientation, and diverse medical needs (e.g., catheter use). Discussion also considered feasibility and usability within primary care settings.

Qualitative data from the working meeting was documented and analyzed using the same inductive content analysis procedures employed in Phase 1. Feedback directly informed further refinement of item wording, response options, and overall measure structure prior to the second Delphi round.

### Phase 3: Delphi Round 2

Following the conclusion of the working meeting and subsequent analysis, the revised screening measure was amended through a second Delphi round conducted via e-mail with members of the expert working group in Phase 2. Revised items were presented to participants, who were asked to indicate agreement or disagreement with the inclusion of each item in the screening measure. Participants who disagreed with item inclusion were asked to provide a rationale for exclusion. Participants were also invited to provide final comments regarding the screening measure, including feedback on the preferred ordering of items. Findings from the second Delphi round were reviewed and interpreted by the same expert working group and informed the final revisions to the screening measure.

Consensus was defined a priori as a clear majority (≥75%) agreement among participants.^33^ Items receiving ≥80% agreement were retained with minor edits if required, items receiving 70–79% agreement underwent more substantial revisions, and items with <70% agreement were removed from the screening measure. Qualitative content analysis of open-text responses followed the same procedures as those used in the initial Delphi round and was used to inform final item refinement.

### Phase 4: Delphi Round 3

The revised questionnaire was subsequently distributed to participants from Phase 2 and 3 in a final, third Delphi round to confirm agreement with the updated questionnaire.

## Results

### Participants

A total of 65 endometriosis experts participated in the first round of the Delphi survey. The mean age of respondents was 40.0 years (SD = 10.6), and the majority identified as women (87.7%). Participants represented three key expert groups. Twenty-four participants identified as PWLE. Clinician participants included 21 gynecologists and 11 pain medicine specialists (specialist care), as well as 11 PCPs. Five additional healthcare professionals (e.g., occupational therapist, psychologist) also participated. Additional participant characteristics are presented in Table 1.

**Table 1.** Characteristics of Delphi Round 1.

Age	40.1 (10.6SD)
**Sex Assigned at Birth**	
Female	58 (89.2%)
Male	7 (10.8%)
**Gender Identity**	
Woman	57 (87.7%)
Man	7 (10.8%)
Gender diverse	1 (1.5%)
**Endometriosis Characteristics Among PWLE**	24 (36.9%)
Age of endometriosis symptom onset	14.8 (5.1SD)
Age of endometriosis diagnosis	25.9 (6.4SD)
Age of PWLE	34.87 (9.96SD)
Gender identity of PWLE	
Woman	23 (95.8%)
Man	0 (0%)
Gender diverse	1 (4.2%)
Method of diagnosis:	
Surgery	17 (70.8%)
Imaging	7 (29.2%)
Unsure	1 (4.2%)
Other	2 (8.3%)
**Primary Care Provider Demographics**	11 (16.9%)
Years practicing as a PCP	14.4 (8.1SD)
Age of PCP	46 (8.79SD)
Gender identity of PCP	
Woman	10 (90.9%)
Man	1 (9.1%)
Gender diverse	0 (0%)
Setting of practice:	
Urban	9 (81.8%)
Rural	2 (18.2%)
Patients seen with endometriosis:	
<25%	7 (63.6%)
25% – 50%	4 (36.4%)
50% – 75%	0 (0%)
>75%	0 (0%)
Age of patients with endometriosis:	
Pediatric (<18)	2 (18.2%)
Young Adult (18–30)	10 (90.9%)
Adult (>30)	7 (63.6%)
**Gynecologist Demographics**	21 (32.2%)
Years practicing as a gynecologist	11.7 (11.05SD)
Age of gynecologist	43.5 (9.02SD)
Gender identity of gynecologist	
Woman	16 (76.2%)
Man	5 (23.8%)
Gender diverse	0 (0%)
Setting of practice	
Urban	21 (100%)
Rural	0 (0%)
Patients seen with endometriosis	
<25%	4 (19%)
25% – 50%	5 (24%)
50% – 75%	6 (28.6%)
>75%	6 (28.6%)
Age of patients with endometriosis	
Pediatric (<18)	5 (24%)
Young Adult (18–30)	16 (76.2%)
Adult (>30)	18 (85.7%)
**Pain Specialist Demographics**	11 (16.9%)
Years practicing as a pain specialist	5.55 (4.95SD)
Age of pain specialist	43.8 (8.01SD)
Gender identity of pain specialist	
Woman	9 (81.8%)
Man	2 (18.2%)
Gender diverse	0 (0%)
Setting of practice	
Urban	8 (72.7%)
Rural	3 (27.3%)
Patients seen with endometriosis	
<25%	5 (45.5%)
25% – 50%	2 (18.2%)
50% – 75%	2 (18.2%)
>75%	2 (18.2%)
Age of patients with endometriosis	
Pediatric (<18)	1 (9.1%)
Young Adult (18–30)	7 (63.6%)
Adult (>30)	9 (81.8%)
**Other Specialist Demographics**	5 (7.7%)
Years practicing as a other specialist	12.00 (9.00SD)
Age of other specialist	45.3 (13.40SD)
Gender identity of other specialist	
Woman	5 (100%)
Man	0 (0%)
Gender diverse	0 (0%)
Setting of practice	
Urban	4 (80%)
Rural	1 (20%)
Patients seen with endometriosis	
<25%	4 (80%)
25% – 50%	0 (0%)
50% – 75%	0 (0%)
>75%	1 (20%)
Age of patients with endometriosis	
Pediatric (<18)	0 (0%)
Young Adult (18–30)	5 (100%)
Adult (>30)	3 (60%)

Interest-holder categories were not mutually exclusive; participants could identify with more than one professional or lived-experience category.

#### Delphi Round 1

All 21 items from the original ENDOPAIN-4D were evaluated for clinical relevance. Mean relevance scores ranged from 7.02 to 9.10 out of 10, with an overall mean of 8.26 across items.

Using the predefined 75% agreement threshold (≥7.5) for inclusion, 18 of the 21 items met criteria for clinical relevance. Three items (Item 6, 19, and 20) did not meet the threshold and were, therefore, flagged for potential removal. See Table 3 for more details.

In addition to quantitative ratings, substantial qualitative feedback was provided. A total of 742 open-text comments were received. Of these, 452 comments related to suggested modifications to item wording, 244 addressed response option wording, and 46 pertained to broader considerations regarding domain coverage and additional feedback.

Consistent with the second stage of review, all items for which modifications were recommended underwent qualitative content analysis. Feedback was grouped into categories related to clarity, inclusivity, feasibility, specificity to endometriosis, and usability in primary care. These findings informed revisions discussed during the working meeting and guided subsequent modifications to item wording, response formats, and overall measure structure.

### Conceptually appropriate domains

Overall, participants agreed that the core domains covered in the ENDOPAIN-4D, namely spontaneous pelvic pain, pain during sexual intercourse, bowel pain and related symptoms, and other associated symptoms, were conceptually appropriate for identifying endometriosis in young adults. Many felt the measure reflected the symptom categories they routinely assess in clinical practice. As one gynecologist noted, “I think it covers every category that I ask. I cannot think of any other category.”

Despite the broad agreement of the relevance of the core domains, some respondents suggested that additional symptom areas could strengthen the screening measure. For example, several participants recommended the screening measure include greater attention to menstrual bleeding patterns and emotional or psychological characteristics. One PWLE shared, “I would include something that talks about irregular bleeding, my biggest symptom (aside from pain) between the ages of 13 and 35 was bleeding for weeks or months on end.”

In response to this feedback, the research team retained the four core domains of the ENDOPAIN-4D but expanded the screening measure to incorporate items addressing menstrual bleeding patterns. Based on participant feedback, we also considered the emotional and psychological impact of symptoms in refining item wording and framing, ensuring that the measure acknowledges the broader lived experience of endometriosis while maintaining its focus as a concise, symptom-based screening instrument suitable for primary care.

### Question clarity

In general, participants felt that the overall clarity of the ENDOPAIN-4D could be improved to reduce potential confusion for patients. One note was in clarifying how pain was described or referred to. For example, in reference to pelvic pain, the ENDOPAIN-4D items refer specifically to pain in the “lower abdomen,” subsequent items refer more broadly to “the pain,” which led some participants to question which type of pain respondents were meant to consider. However, given the systemic nature of endometriosis, PWLE articulated that even pelvic pain could not be limited solely to lower abdomen. This was further echoed by clinicians. As one gynecologist asked, “What pain are they describing? Their dysmenorrhea? Their non-cyclic flares, etc?”

Participants also commented on the use of specific pain descriptors. Some items included sensations such as stabbing, sharp, or deep pain, or combined multiple sensations within a single question. Several respondents indicated that these descriptors might not resonate with all individuals and could contribute to misinterpretation. As one PWLE explained, “Not everyone with endometriosis experiences this quality of pain, i.e. some may describe their pain as burning, aching, throbbing, etc.” Another noted,

I wonder if setting up a definition of pain at the beginning of the [screening measure] may be beneficial. Many people who have chronic pain, do not actually refer to it as pain … sometimes it is more ‘pressure, or discomfort, or heat or another feeling. [PCP + Pain Specialist].

In response to this feedback, the research team revised the wording of items to consistently specify the type and location of pain being assessed and incorporated broader, more inclusive language to reflect the range of sensations patients may experience. Where appropriate, clarifying statements were added to reduce ambiguity and improve comprehension. Further, we simplified pain descriptors, avoided specific or restrictive terminology, and ensured that items allowed variability in how pain is experienced and described.

Finally, participants identified opportunities to improve overall flow and consistency, including clarity around timeframes (e.g., six months versus one year), minor grammatical issues, and order of the questions being asked. Accordingly, the team standardized timeframes across items, and conducted a comprehensive review to improve grammar, readability, and overall flow of the measure.

### Response option clarity

Many participants noted that the response scale did not align well with the question prompts, leading to confusion. For example, participants highlighted concerns about language inconsistencies with the 0–10 rating scale used throughout the ENDOPAIN-4D. The wording of the scale varied across items, shifting from “how would you rate what you feel” to “how would you rate your discomfort with regard to this problem” to “how would you rate your pain at its worst.” These changes were perceived as potentially confusing. As one participant noted, “I worry about the multiple changes to the scale, as it may be missed by survey respondents. It may benefit to keep the scale as consistent as possible” [Gynecologist + Pain Specialist].

There was general agreement that a simpler response format would be more appropriate for a screening measure. Several participants suggested that a straightforward yes/no format would be sufficient to identify individuals who may warrant further assessment for endometriosis. As one pain specialist stated, “0 to 10 scale may not be appropriate as they’re already identifying it as unbearable (often then correlating with10/10 pain in clients).” Another suggested that prompts might also be beneficial.

The scale seems incongruent with a ‘yes’ … I would use responses along the lines of ‘rarely, sometimes, most of the time, always’ if that. A screening tool should be simple, easy to administer, and prompt the more detailed questions that should follow if the screen is positive. [Gynecologist].

In response to this feedback, our team revised the response options to prioritize simplicity and consistency and align response options more closely with question prompts. This resulted in primarily adopting *yes/no* formats and, where appropriate, *never to always* scales.

### Item redundancy

Participants highlighted that several items in the original ENDOPAIN-4D repeated similar concepts. For example, items 7, 8, and 19 centered around pain radiating from the pelvis to other areas of the body. One PWLE reflected, “would the reference to sciatica be different than asking about low back, leg or hip pain earlier?” Items 3, 6, and 9 that focus on pain interference and disability were also highlighted as conceptually similar, “if the pain is limiting their everyday activity, that is enough information usually” [PCP]. While these items received a high score for relevance and were deemed conceptually important for endometriosis, our team opted to improve clarity and reduce redundancy by grouping concepts into single items.

### Simplify for the Canadian young adult audience

The focus of our Delphi was to adapt the ENDOPAIN-4D for a Canadian young adult population. As such, many respondents highlighted phrases, concepts, or words that may not be applicable to our audience. For example, a pain specialist noted, “I don’t regularly use ‘passing a stool.’ It does not seem like a term young adults would use. Possibly ‘going to the bathroom [or] painful bowel movements.” Further, the participants noted that areas of the body or conditions, may not be universally understood (e.g., sciatica).

Some concepts, such as disability and sexual activity, were identified as being too vague. One gynecologist asked, “Do you think disabling is a word easy enough to understand for most people? Maybe I would add in parentheses ‘has a negative impact on work/school/social activities.” Respondents also noted that the measure should contain Canadian spelling. As a result, our team focused on ensuring the screening measure would be more applicable to our audience by updating the language to reflect the audience, including by updating the terms used to a grade eight reading level. If any complex phrases were required, we would feature a definition in lay terms.

#### Working meeting

The working meeting reviewed the Delphi feedback and engaged in further expert deliberation, resulting in final questionnaire revision decisions. The characteristics of the working meeting and Delphi Round 2 are captured in Table 2. A summary of these decisions is presented in Table 3. The updated question items and corresponding response options are provided in Table 4.

**Table 3.** Summary table.

Item Number	Question	Ratings	Decision
**DOMAIN 1: Spontaneous Pelvic Pain**			
Item 1	Over the past few months, have you regularly suffered from: Severe, violent pain in **the lower abdomen**, during your period.	Median = 10 (IQR 8–10) Mean = 8.71 (1.86SD)	**Modify the question**
Item 2	Over the past few months, have you regularly suffered from: Severe, violent pain in **the lower abdomen**, between periods.	Median = 10 (IQR 8–10) Mean = 8.41 (2.30SD)	**Modify the question**
Item 3	The pain is very intense, it **is violent, cannot be ignored, is unbearable.**	Median = 9 (IQR 6–10) Mean = 7.90 (2.49SD)	**Remove question**
Item 4	**As the years go by**, the pain is getting worse.	Median = 9 (IQR 6.5–10) Mean = 8.05 (2.52SD)	**Modify the question**
Item 5	The pain comes a few days **before your period** and/or continues for a few days **after your period.**	Median = 9 (IQR 8–10) Mean = 8.51 (2.01SD)	**Modify the question**
Item 6	The pain **comes and goes suddenly**; it is a **stabbing pain**.	Median = 8 (IQR 5–10) Mean = 7.27 (2.61SD)	**Remove question**
Item 7	The pain spreads to your **back**, in the **lower back area.**	Median = 9 (IQR 7–10) Mean = 8.25 (2.26SD)	**Merge Item 7 and 8**
Item 8	The pain spreads to your **legs** and **hips**.	Median = 8 (IQR 7–10) Mean = 7.78 (2.51SD)	**Merge Item 7 and 8**
Item 9	The pain becomes **disabling** for **everyday activities**	Median = 10 (IQR 9–10) Mean = 9.10 (1.66SD)	**Merge Item 9 and 10**
Item 10	The pain prevents you from **standing, walking, moving**	Median = 9 (IQR 7–10) Mean = 8.30 (2.28SD)	**Merge Item 9 and 10**
**DOMAIN 2: Pain During Sexual Intercourse**			
Item 11	Severe, sharp and **deep internal pain** during intercourse	Median = 10 (IQR 8–10) Mean = 9.02 (1.66SD)	**Modify the question**
Item 12	Pain felt in **certain positions** during intercourse	Median = 9 (IQR 7.5–10) Mean = 8.09 (2.29SD)	**Remove question**
Item 13	Pain that **upsets, prevents** or **interrupts** sexual intercourse	Median = 9 (IQR 8–10) Mean = 8.44 (2.10SD)	**Modify the question**
**DOMAIN 3: Bowel Pain and/or Symptoms**			
Item 14	Pain when **passing a stool; painful bowel movements** particularly during your period	Median = 10 (IQR 8–10) Mean = 9.07 (1.40SD)	**Modify the question**
Item 15	**Spasms, cramps, bowel pain** before a bowel movement, particularly during your period	Median = 10 (IQR 8–10) Mean = 8.69 (1.96SD)	**Remove question**
Item 16	**Diarrhea and/or constipation**, particularly during your period	Median = 9 (IQR 7.5–10) Mean = 8.09 (2.75SD)	**Modify the question**
**DOMAIN 4: Other Symptoms**			
Item 17	**Difficulty and/or pain when urinating** particularly during your period	Median = 9 (IQR 7–10) Mean = 8.29 (2.38SD)	**Modify the question**
Item 18	Pain in the **bladder**, when you **want to urinate**, or when **holding back**, particularly during your period	Median = 9 (IQR 7–10) Mean = 8.07 (2.19SD)	**Remove question**
Item 19	**Sciatica** particularly during your period	Median = 8 (IQR 6–10) Mean = 7.40 (2.72SD)	**Remove question**
Item 20	Pain in the **right shoulder**, or **under right rib cage** particularly during your period	Median = 7.5 (IQR 5.75–10) Mean = 7.02 (2.90SD)	**Remove question**
Item 21	Difficulty **becoming pregnant, failure to conceive despite trying** for several months or years	Median = 10 (IQR 9–10) Mean = 9.04 (1.75SD)	**Modify the question**

**Table 4.** Endometriosis screening measure items, response options, and Delphi Round 2 consensus agreement.

Item number	Item	Response option	Proportion agreed to include			
1	How old were you when you had your first period?	Free text: __________________ ☐ I don’t remember	*75%*			
**DOMAIN 1: Pelvic Pain**						
2	Within the past six months, have you had an episode or multiple episodes of pelvic pain?	If yes, how would you rate your pain at its worst? (0–10) ◻ Yes ◻ No	*94%*			
3	Did this pelvic pain start with your first period?	◻ Yes ◻ No ◻ I do not know/I cannot remember ◻ Not Applicable (e.g., I don’t have pelvic pain)	*81%*			
4	Since the first time you felt pelvic pain, has your pelvic pain gotten worse?	◻ Yes ◻ No ◻ Not Applicable (e.g., I don’t have pelvic pain)	*88%*			
5	Do you notice a pattern with your pelvic pain? For example, some people notice their pelvic pain occurs at different times of their menstrual cycle (e.g., menstruation/your period or ovulation/mid cycle).	☐ Yes ☐ No ☐ I do not know ◻ Not Applicable (e.g., I don’t have pelvic pain)	*100%*			
6	Does the pain in your pelvic area spread to your lower back, hips, buttocks, or legs? (please refer to the diagram below)	◻ Never ◻ Rarely ◻ Sometimes ◻ Often ◻ Always	*88%*			
7	How often does your pelvic pain make it difficult to go to school, work, do social activities, or fun activities as much as you would like?	◻ Never ◻ Rarely ◻ Sometimes ◻ Often ◻ Always	*88%*			
8	Does it hurt to insert a tampon or menstrual cup (i.e., DivaCup)?	◻ Never ◻ Rarely ◻ Sometimes ◻ Often ◻ Always ◻ Not Applicable I don’t use these items ◻ Tried but was too painful	*94%*			
**DOMAIN 2: Pain During Sexual Intercourse**						
9	Have you had pelvic pain with sexual vaginal penetration (for example, something inserted inside the vagina like a penis, finger, or object)?	☐ Yes ☐ No ☐ Not Applicable (e.g., I have not had any kind of sexual vaginal penetration) ☐ Prefer not to answer	*88%*			
10	How often does your pelvic pain make it difficult for you to engage in sexual vaginal penetration (for example, something inserted inside the vagina like a penis, finger, or object)?	◻ Never ◻ Rarely ◻ Sometimes ◻ Often ◻ Always ◻ Not Applicable (e.g., I have not had any kind of sexual vaginal penetration) ◻ Prefer not to answer	*81%*			
**DOMAIN 3: Bowel Movements (pooing)/Urination (peeing)**						
11	Over the past six months, have you had pelvic pain when pooping (bowel movement)?	◻ Yes ◻ No	*88%*			
12	Is this pelvic pain when pooping (bowel movement) worse when you are on your period?	◻ Yes ◻ No ◻ Not applicable (e.g., I don’t have a period, I don’t have pelvic pain when pooping)	*94%*			
13	Do you fear/ feel anxious about pooping (bowel movement) because of the pain?	◻ Never ◻ Rarely ◻ Sometimes ◻ Often ◻ Always ◻ Not applicable (e.g., I don’t have pelvic pain when pooping)	*75%*			
14	Over the past six months, have you had diarrhea (loose poop) or constipation (difficulty pooping)?	◻ Never ◻ Rarely ◻ Sometimes ◻ Often ◻ Always	*75%*			
15	Is your diarrhea (loose poop) or constipation (difficulty pooping) more bothersome just before or during your period?	◻ Yes ◻ No ◻ Not applicable (e.g., I don’t have a period, I don’t have diarrhea or constipation)	*88%*			
16	Over the past six months, have you had pelvic pain when peeing (urinating)?	◻ Yes ◻ No ◻ Not applicable (e.g., I have a catheter)	*94%*			
17	Is the pelvic pain while peeing (urinating) worse when you are on your period?	◻ Yes ◻ No ◻ Not applicable (e.g., I don’t have a period, I have a catheter)	*94%*			
18	Do you fear/ feel anxious about peeing (urinating) because of the pain?	◻ Never ◻ Rarely ◻ Sometimes ◻ Often ◻ Always ◻ Not applicable (e.g., I don’t have pelvic pain when peeing)	*100%*			
19	Do you notice that you pee (urinate) more often at certain times of the month? For example, some people notice more frequent peeing at different times of their menstrual cycle (e.g., menstruation/your period or ovulation/mid cycle).	◻ Yes ◻ No	*75%*			
**DOMAIN 4: Fertility**						
20	Have you been trying to become pregnant for 12 months in a row or for longer, but have not become pregnant?	☐ I have been trying and NOT become pregnant ☐ I have been trying and HAVE gotten pregnant ☐ I have not tried to become pregnant, but I have had regular vaginal sexual intercourse without using birth control and have NOT become pregnant ☐ I have not tried (e.g., used birth control) to become pregnant ☐ Prefer not to answer	*81%*			
**DOMAIN 5: Other**						
21	Do you experience heavy periods (i.e., you use more than one pad/hour for several hours straight, pass blood clots, **or** experience blood flow that interrupts your daily activities)?	◻ Never ◻ Rarely ◻ Sometimes ◻ Often ◻ Always ☐ Not Applicable (e.g., I don’t have a period)	*75%*			
22	Have you ever used birth control/ hormonal medications (like the pill, patch, IUD, or injection or contraception) to help with your period pain, to make your period more regular, or easier to manage?	☐ Yes, I *currently* use something ☐ Yes, I used it *in the past* ☐ No ☐ I’m not sure ☐ I prefer not to answer	*94%*

**Table 2.** Characteristics of the working meeting, Delphi Round 2 and 3.

Event	Number of participants	Age	Gender Identity	PWLE	Primary Care Provider	Gynecologist	Pain Specialist	Other Specialist
Delphi Round 1	65	40.1 (10.65SD)	57 Woman 7 Man 1 Gender Diverse	24	11	21	11	5
Working Meeting	15	46.8 (11.1SD)	13 Woman 2 Man	5	2	3	6	0
Delphi Round 2	16	46.9 (10.6SD)	14 Woman 2 Man	6	2	4	7	0
Delphi Round 3	18	47.6 (10.6SD)	15 Woman 3 Man	6	3	4	8	0

Interest-holder categories were not mutually exclusive; participants could identify with more than one professional or lived-experience category.

#### Delphi Round 2

In Delphi Round 2, all items achieved consensus (see Table 4), with agreement ranging from 75% to 100% (mean agreement = 86%). Five items (1, 13, 14, 17, and 21) fell below 80% agreement and were, therefore, further revised based on qualitative feedback.

Item 1 is concerned with age at menarche. It was previously located at the end of the screening measure (question number 21). Although consensus was achieved, it was not noted to be of clinical significance. Participants recommended relocating this item within the survey and retaining it for contextual purposes. However, its inclusion is as a non-scored item that would not contribute to the overall screening score. *Final wording: How old were you when you had your first period?*

Items 13 and 14 relate to bowel movements, including fear of bowel movements due to pain and diarrhea and/or constipation frequency, respectively. For item 13, agreement was relatively lower and some participants questioned the diagnostic specificity of the item. However, as the language was updated to reflect if patients “fear/feel anxious about pooping (bowel movements) because of the pain,” other participants emphasized how the item could capture the patient perspective and the lived experience of pain-related avoidance. The item was retained in the final version. *Final wording: Do you fear/feel anxious about pooping (bowel movements*) *because of the pain?* For item 14, minor revisions were made to improve clarity, accessibility, and consistency in interpretation. *Final wording: Over the past six months, how often have you had diarrhea (loose poop) and/or constipation (difficulty pooping/hard poop?).*

Item 17 is related to urinary frequency across the menstrual cycle. Consensus was achieved. Minor modifications were made to improve question flow and response structure, including changing response options from a frequency scale to a dichotomous (yes/no) format. *Final wording: Do you pee (urinate) more often at certain times? For example, some people notice more frequent peeing at different times of their menstrual cycle (e.g., menstruation/your period or ovulation/mid-cycle).*

Lastly, item 21 is related to heavy menstrual bleeding. Based on participant feedback, response options were revised from a frequency scale to a yes/no format to improve clarity and reduce ambiguity. *Final wording: Do you experience heavy periods (i.e., you use more than one pad/hour for several hours straight, pass blood clots, or experience blood flow that interrupts your daily activities)?*

#### Delphi Round 3

In Delphi Round 3, the revised questionnaire was redistributed to panel members for final review and rating. All items achieved greater than 80% agreement, meeting the predefined threshold for consensus. The expert panel endorsed the final set of questions without further recommended revisions, concluding the Delphi process and establishing the finalized endometriosis screening measure.

## Discussion

This study addressed an important gap in endometriosis identification by adapting an existing symptom-based screener for Canadian young adults in primary care. Through a four-phase Delphi-informed co-development process, we developed the Screening for Early Endometriosis Detection (SEED), a 22-item measure designed to be developmentally appropriate, gender-inclusive, trauma-informed, and feasible for frontline clinical use. Given the longstanding barriers to timely endometriosis diagnosis among women and gender-diverse individuals, we revised a validated endometriosis screening measure (ENDOPAIN-4D),^32^ which was originally validated in the French context and among women with advanced disease who were older than our target population. The resulting screening measure includes consensus-informed items reflecting symptom domains considered relevant for identifying possible endometriosis in young adults within Canadian primary care.

The development of SEED is consistent with Canadian priorities to advance women’s health. Delays in endometriosis diagnosis are shaped by barriers at both the individual and health-system levels. Symptoms may be normalized^35,36^ even when they are severe and disruptive, while patients may also be dismissed or encounter limited provider knowledge of endometriosis.^4^ These barriers are shaped, in part, by stigma surrounding women’s health issues. Chronic pain and pelvic pain in particular lacks appropriate research representation and, as a result, remains a top priority for many calls to action.^37,38^ Endometriosis represents a key priority for investigation and has been described by researchers as a “missed disease” and a “modern epidemic.”^39^

Diagnostic delay arising from limited awareness and recognition among healthcare providers, as well as stigma toward female reproductive health, has resulted in excess disease burden for women and gender-diverse individuals living with both diagnosed and undiagnosed endometriosis. The 24 PWLE included in this study had a mean symptom onset of 14.8 years and a mean age of diagnosis of 25.9 years. This experience of delayed diagnosis is similar to the average Canadian reported delay of approximately 11 years. Recent research also suggests that delays may be worsening in some settings, with a UK survey reporting that endometriosis diagnosis times increased by 10 months between 2020 and 2023.^40^ For trans and gender-diverse populations, who face further healthcare inequities due to barriers to care and limited access to treatment, delays in receiving both adequate treatment and an endometriosis diagnosis may be even greater.^41^ Thus, it was a priority for the team to highlight gender-inclusive language and perspectives when revising the screening measure.

Our findings are consistent with the broader cross-cultural adaptation literature, which emphasizes that preserving conceptual equivalence across settings may still require substantial item-level revision.^42,43^ Although the core ENDOPAIN-4D symptom domains remained relevant for identifying endometriosis in young adults, several items required removal, consolidation, or refinement. This aligns with prior guidance that adaptation should extend beyond translation to include semantic clarity, contextual appropriateness, readability, and respondent experience.^27,42^ The depth of qualitative feedback in our study underscored that high quantitative agreement alone was insufficient for retaining items, particularly when participants identified wording that was confusing, uncommon in the Canadian context, or too complex for the intended reading level. Similar issues have been reported in endometriosis-specific adaptation work, where local testing has been needed to clarify terminology and improve comprehension.^44,45^ Together, these findings suggest that adapting symptom-based screening measures for new populations requires not only preservation of core constructs, but also iterative refinement to ensure usability, inclusivity, and fit with the target clinical context.^43,46^

Dissemination and implementation represent key considerations in the development of screening measures. SEED is intended for use in primary care, where many patients first present with symptoms and where referral to gynecological services is often initiated. However, uptake in primary care depends on more than the strength of the measure itself. Utilization is influenced by factors such as awareness, familiarity, confidence, and healthcare provider training.^47,48^ As such, the clinical setting, who is administering the measure, ease of scoring, method of administration, and accessibility of the measure were considered in the development of SEED and remain important priorities for future work. Importantly, the inclusive and non-stigmatizing language established in the development of SEED may itself support implementation by helping patients feel respected and safe while completing the measure, and by building trust that their symptoms and honest reflections will be taken seriously; this is consistent with literature showing that stigmatizing language in healthcare can negatively affect patient perception of care and influence subsequent clinical decision-making.^49–51^ Consideration must also be given to practical barriers such as copyright, financial resources, dissemination strategy, and integration into paper-based or electronic systems, as feasibility, implementation cost, appropriateness, and sustainability are recognized determinants of whether innovations are successfully adopted in routine care. While symptom-based measures such as SEED may improve the identification of people at higher risk of endometriosis, equal attention is required to understand how to support their adoption, sustainability, and impact in real-world clinical settings.^52,53^

### Limitations

While this study aimed to be inclusive of diverse perspectives in the development of the SEED, and included representation across clinical roles and lived experience, the study design is not without limitations. During the Delphi rounds and working group components of the study, the research team maintained anonymity to reduce any conscious or unconscious influence from other participants, including potential power dynamics that might exist between healthcare providers and PWLEs. It is generally not possible to control all group biases during meetings, however, the research team attempted to limit the impact of any biases that might exist through multiple avenues, including various methods of collecting feedback from participants (e.g., participants were able to anonymously comment on a whiteboard, they were able to send both public and private messages in the Zoom chat, they were able to send reaction emojis, like the thumbs up or down, and were allowed to orally comment). Additionally, the placement of the working meeting between Delphi rounds one and two allowed participants to further revise items without conformity and agreement biases that may be present within small groups. At the same time, because participants were recruited through professional and community networks and the working group was purposively sampled, the panel may have overrepresented individuals already engaged in endometriosis care, research, or advocacy, which may limit generalizability. Some perspectives may also have been underrepresented, including those of rural clinicians, francophone populations, younger individuals with lived/living experience, Indigenous and racialized communities, newcomers, trans and gender-diverse people, and individuals with lower health literacy. Although the smaller working group supported continuity and detailed co-development of the substantially revised measure, the final consensus reflects this selected subgroup rather than the full Delphi Round 1 respondents. In addition, this study focused on the development of an English-language version of the measure and, therefore, did not assess linguistic or cultural equivalence across other languages used in Canada.

An additional limitation is that the consensus achieved between participants on the SEED items and their wording does not equal validity. Future studies will need to assess the validity (psychometric performance and diagnostic accuracy within a broader gender, geographically, ethnically, and racially diverse sample) of the SEED before widespread implementation.

## Conclusions

This four-phase study to refine the ENDOPAIN-4D for a Canadian young adult population resulted in the development of a 22-item screening measure formally called the Screening for Early Endometriosis Detection (SEED). Five core domains were identified: 1) pelvic pain, 2) pain during sexual intercourse, 3) bowel movements (pooing)/urination (peeing), 4) fertility, and 5) other symptoms. Further research to assess the validity of the SEED is required before widespread adoption. A screening measure focused on young Canadian adults may support earlier recognition of endometriosis and, pending further validation and implementation work, could contribute to improved care pathways and patient outcomes.

## Data Availability

Data is available upon request.
